# Competence assessment rubric in the Physiotherapy Practicum

**DOI:** 10.1371/journal.pone.0264120

**Published:** 2022-02-25

**Authors:** Noa Lola Martiáñez-Ramírez, Consolación Pineda-Galán, María Rodríguez-Bailón, Rita-Pilar Romero-Galisteo

**Affiliations:** 1 Department of Physiotherapy, La Salle Higher Education Center, Autonomous University of Madrid, Madrid, Spain; 2 Department of Physiotherapy, Faculty of Health Sciences, University of Málaga, Málaga, Spain; Mugla Sitki Kocman Universitesi, TURKEY

## Abstract

**Introduction:**

Competence-based learning must be integrated into the practical development of Physiotherapy. Teamwork, interpersonal relations, analytical skills or critical/clinical thinking are some examples of internationally recommended competences in this kind of university studies. Therefore, there is a need to evaluate this learning in Physiotherapy through valid tools that facilitate this task.

**Objective:**

To analyze the psychometric properties according to competences in Clinical Practices (RECOPC-FIS II) in order to assess 14 transversal or universal competences of under-graduate students in the Physiotherapy degree.

**Methods:**

A validation study was conducted with 197 students in the 3^rd^ and 4^th^ year of the Physiotherapy degree and 202 clinical tutors who assessed these students using the RECOPC-FIS II. Different psychometric properties were analyzed: factor structure, internal consistency and sensitivity to change.

**Results:**

The RECOPC-FIS II has a high internal consistency. Its 14 items saturate in a single factor. Regarding the sensitivity to change, the rubric showed higher scores in the Practicum of the 4^th^ year with respect to that of the 3^rd^ year, reaching significant differences in all of them.

**Conclusion:**

The RECOPC-FIS II is a valid and reliable instrument to assess the transversal competencies of undergraduate students of Physiotherapy during their clinical practice. Therefore, it is intended to facilitate the acquisition of essential skills for the development of their professional career. The flexibility of this tool would allow its adaptation to other health science courses.

## Introduction

Recently, there has been a paradigm shift in higher education with an increased focus on competence training. Competences are dynamic combinations of knowledge, procedures and attitudes [1]. They represent what those who undertake a program must be able to perform once such program is completed.

Competence-based learning must be integrated into the practical development of Physiotherapy and, due to their nature, competences can only be reached in the final stages of a training process [2–4]. One of the main characteristics of competences is that they can be improved through training, interventions or experience [5–7]. It has also been demonstrated that competences correlate with different academic and performance variables, such as procrastination, self-efficacy and critical thinking, among others [8]. In this way, the use of this type of tools in higher education will ensure and improve the quality of the teaching-learning processes [9].

Quality improvement has been advocated in the European higher education area [10]. Competency assessment is an important task that many agencies have used to accredit, audit, or certify institutions. Both internationally and nationally, there are several organizations within each country that refer to the guidelines that will guide the curricula and training programs for health sciences students, specifically in Physiotherapy, for the development of competences [11–15].

Within competences, we can consider the universal or transversal competences, which are common to most degrees, although with a different incidence, and contextualized in each degree course [16]. These types of competences are defined as the generic [16] aspects of knowledge, abilities and skills that any higher education student should have [2]. Universal or transversal competences can be classified into instrumental, interpersonal and systematic. Instrumental competencies are those that have a media function and are aimed at the acquisition of cognitive, methodological, technological and linguistic skills, as well as academic achievement (e.g., completing the clinical history). Interpersonal competences are those related to the development of personal skills and social relationships, including those skills related to the way of communicating and accepting feelings and emotions that express generosity towards others, cooperation and social interaction (e.g., interdisciplinary teamwork and ability to communicate with experts from other areas, among others). Lastly, systemic competences are those that require the integration of the entire system and, therefore, demand a prior acquisition of interpersonal competences (e.g., ability to apply knowledge in practice) [17].

Despite the fact that competence training has been greatly improved, using competences to guide curriculum development has proved problematic [18]. In the field of health science in universities, modifications are required to meet the new socio-sanitary needs, ensuring the development of competence profiles in students, oriented towards good professional and human performance [19].

Among all the courses of higher education degrees in health sciences, one of the most important is the Practicum (clinical experience) [20, 21]. Students should carry out clinical practices in which they will integrate previously acquired knowledge, applying it to specific clinical cases. Therefore, it is during the practicum that all the transversal competences will be developed. It is also a specific case in which competences are assessed in a relevant way, and the context gains special importance. “History taking competence" would be a good example to illustrate this idea: history taking, filling in or recording is used in the context for students’ learning in the clinical setting. The management of the Practicum implies an important joint work between the university and the healthcare system. This partnership implies an adaptation and integration of the competences learned in the academic field, taking the set of learning outcomes specific to each academic course as a reference.

Due to the relevance of competence training included in the Practicum, it is essential to ensure its assessment, even though it is a challenge for any teacher involved in the training process [22], particularly for clinical tutors. In clinical practice, competence assessment is based on the direct tutors’ observation of students’ performance providing care to real patients in the healthcare environment. In this way, the assessment criteria should be explicit, specific, and related to the professional profile [23, 24].

To that end, it is necessary to create valid and reliable competence assessment tools to evaluate performance in the authentic practice environments related to health sciences [25]. One of the tools that facilitates the assessment of students’ performance is the ‘rubric’ [26–30]. Rubrics, which include concise achievement criteria, rating scales and a description of the expected performance at each level [31], facilitate individual ratings adapted to the competences of each student. Taking into account the elements that characterize the use of rubrics in the qualification of the Practicum would provide an effective solution to determine the levels of competence achievement. In this way, biased judgments made by clinical tutors would be avoided [32].

In addition, in the case of Practicums, the use of rubrics is an essential element [33], since the students are distributed in different healthcare institutions. In this sense, e-rubrics have been proposed to facilitate communication between the different institutions involved, which are several and with very different characteristics and operations regarding the Practicums [26, 34]. Ensuring good understanding and fluid communication with all those involved should be a fundamental priority.

Despite their pragmatism, there are scarce rubrics published in the international literature for the assessment of Physiotherapy Practicums [12, 35–37]. Thus, some tools can be found, such as the one proposed by Dalton et al. [12, 35], in which the instrument named Assessment of Physiotherapy Practice (APP) presents ICC values between 0.72 and 0.92 and evidence of construct validity provided by the Rasch analysis. On the other hand, Dogan et al. [36], propose using a rubric in the Physiotherapy practical examination, where they only analyze the degree of agreement between raters (kappa = 0.47 p<0.01) when using a rating scale by categories. In contrast, in the tool proposed by Torres-Narváez et al. [37], named Measurement Tool for Clinical Competencies in PT(MTCCP), content validity is analyzed using the Content Validity Index (CVI >0. 8), internal consistency using Cronbach’s alpha coefficient = 0.982 and construct validity using an exploratory factor analysis, in which the Kaiser-Meyer-Olkin values were acceptable (KMO>.8). Even so, none of them incorporates specific achievement levels for each of the assessed competences. There are other rubrics for assessing the practical content of specific physiotherapy courses, but not in clinical contexts [36] or whose psychometric properties, based on validity evidence and reliability data reported, are rather limited [38].

For this purpose, the RECOPC-FIS I (Rubric for Competence assessment in Clinical Physiotherapy Practices) was created in Spain [24] through a multicenter study conducted in the community of Madrid, with the participation of eleven universities and thirteen healthcare centers. Based on this proposal, it was possible to improve the internal validity of the tool through a committee of experts, who proposed expanding the number of items to be assessed, thus generating the RECOPC-FIS II.

The RECOPC-FIS II includes 14 items related to the most relevant competences (transversal and specific), which guide the student towards a competence itinerary that responds to the requirements of the curriculum. It evaluates oral and written communication, teamwork, interpersonal relationships, analysis and synthesis capacity, critical/clinical thinking, decision making, autonomous learning, initiative and entrepreneurship, and the capacity to adapt to new situations, among others (see S1 Appendix). However, although this tool was adapted to the curriculum of the University of Málaga and agreed on with a group of experts, it still has not been validated in students.

The main objective of the present study was to assess the psychometric properties of the RECOPC-FIS II, in order to have a valid and reliable tool for the assessment of transversal competences in the Practicums of Physiotherapy university programs.

## Materials and methods

A total of 202 tutors completed the RECOPC-FIS II regarding the clinical practices carried out by 197 students of the degree of Physiotherapy of the University of Málaga (112 were woman: 56.85%): of which 61 were evaluated in P1 (3^rd^ year) and P2 (4^th^ year), 6 students only in P1 and 130 in P2. The response rate was 100% (202/202). The tutors were all physiotherapists working in public or private centres, in the specific services that each rotation required (e.g. geriatrics, neurology, paediatrics, etc.) and with at least 1 year of clinical experience. Clinical tutors were also required to sign a specific collaboration agreement with the university from which the students they were tutoring came.

The tool was administered by the clinical tutors of the degree of Physiotherapy of the University of Málaga. Through an online questionnaire, a competence assessment was carried out in the 2 subjects that make up the Practicum of the degree of Physiotherapy: P1 and P2, during the academic years 2017–2018 and 2018–2019. Different tutors evaluated the students in P1 and P2. The tutors did not know the qualifications of other practicums of the students who were doing the practices with them.

The clinical tutors were trained in the use of RECOPC- FIS II. The tutors had to grade each of the competences at the end of the practice period of the student.

As part of the administration of RECOPC-FIS II, the students were also informed about the assessment system, showing them the rubric and letting them know that, halfway through the practice period, the clinical tutor would give them feedback on the development of their practice and any suggestions for improvement to ensure the correct development of the competences. The clinical tutor must conduct this feedback procedure, which is considered of utmost importance, since the student is not always aware of the aspects that he/she needs to improve, thus granting an essential role to improvement attitude and development.

All the participants signed an informed consent. The study was approved by the Ethics Committee of the University of Málaga (CEUMA 7-2020-H).

RECOPC-FIS II is a transversal competence assessment tool that evaluates 14 competences, transformed into 14 evaluable items or criteria in this rubric for education practice, which are: C1, identification of the dysfunction or pathology; C2, physiotherapy diagnosis (assess the functional state of the patient/user); C3, therapeutic objectives; C4, integral treatment; C5, treatment planning; C6, evaluation of the results; C7, technical and manual skills in the application of therapies; C8, responsibility regarding the attention given to the patient and suitability of the clinical interventions; C9, clinical reasoning; C10, interpersonal relationships; C11, oral communication with the patient, relatives and interdisciplinary team; C12, team work; C13, disease prevention and promotion of health; and C14, autonomous learning (see S1 Appendix).

The fourteen evaluable criteria of RECOPC-FIS II correspond to competences related to the learning of conceptual contents and the mastering of procedural and attitudinal contents. Some of the items evaluate several transversal competences at the same time.

Furthermore, RECOPC-FIS II includes four achievement levels, to avoid intermediate values. The tutor had to select one of the four levels based on the knowledge, involvement in learning and autonomy of the student, and then give a numerical grade. For each competence, the questionnaire includes specific information about each achievement level (Table 1).

**Table 1:** Example of achievement levels for the competence “therapeutic goals”.

Achievement Level	Information on each Achievement Level
**From 0 to 4**	He/she does not set clear, coherent and feasible goals. He/she does not take into account the patient’s own situation and expectations.
**From 5 to 6**	He/she set the goals in a coherent way, but sometimes he/she lacks efficiency in his/her approaches. He/she does not always take into account the goals set by the patient, his/her situation and the stage of the pathology.
**From 7 to 8**	He/she establishes the goals in a coherent and efficient way, prioritizing the needs of the patient. Considers the goals set by the patient, his/her situation and the stage of the pathology.
**From 9 to 10**	He/she points out a comprehensive and hierarchical approach to the biopsychosocial needs of the patient, always taking into account their goals and expectations.

For the grading of the students and for this validation study, we gathered both the ratings given by the tutors to each student for each item of the rubric and the total mean of all the items for each Practicum (P1-P2).

With the aim of analyzing the validity of the assessment instrument, an exploratory factor analysis (EFA) was carried out with all the items, in order to determine the accuracy of the measurement with respect to what was evaluated. Prior to the EFA, we conducted the Bartlett’s sphericity test, as well as the Kaiser-Meyer-Olkin test to determine the adequacy of carrying out this analysis. The internal consistency was calculated using Cronbach’s alpha. For these analyses, the sample consisted of 197 evaluations. Of the students who had been evaluated in both P1 and P2 (n = 61), the average of each of the competencies was calculated.

Lastly, with the aim of analyzing the instrument’s sensitivity to change, we conducted the Student’s T-test for related samples, comparing each item of the corresponding scale of P1 and P2, for a group of students. Based on their knowledge and experiences, the P2 students were expected to obtain higher grades than the P1 students.

All the analyses were carried out using IBM SPSS Statistics v.21.0. All relevant data are within the manuscript and its Supporting Information files (S1 Dataset).

## Results

The mean score of almost all the items was around 8 points. Table 2 shows the means of the ratings obtained in each group (P1, n = 67 and P2, n = 191) for each item.

**Table 2:** Mean and standard deviation of the ratings for each item of RECOPC-FIS II in each group.

Item	C1		C2		C3		C4		C5		C6		C7	
**Group**	P1	P2	P1	P2	P1	P2	P1	P2	P1	P2	P1	P2	P1	P2
**Mean**	7.97	8.34	8.20	8.30	8.14	8.42	8.12	8.52	8.05	8.46	8.05	8.32	8.32	8.60
SD^a^	1.10	0.77	0.72	0.76	0.75	0.73	0.82	0.73	0.73	0.77	0.77	0.74	0.78	0.81
**Item**	C8		C9		C10		C11		C12		C13		C14	
**Group**	P1	P2	P1	P2	P1	P2	P1	P2	P1	P2	P1	P2	P1	P2
**Mean**	8.26	8.61	8.23	8.41	8.66	8.93	8.48	8.82	8.49	8.72	8.04	8.49	8.16	8.47
SD^a^	0.84	0.75	0.80	0.72	0.92	0.81	0.98	0.77	0.86	0.81	0.86	0.79	0.86	0.92

Preliminary analyses showed that the data were appropriate for an EFA [Kaiser–Meyer–Olkin (KMO) measure of sampling adequacy = 0.97; Bartlett’s sphericity test (χ2/gl = 3363.52/91. p < .001). All the items were grouped in a single factor, which explained 76.93% of the variance. The internal consistency of the tool obtained with Cronbach’s alpha was 0.976.

With the aim of analyzing the sensitivity to change of RECOPC-FIS II, 61 students were selected, who were evaluated both in P1 and then in P2. As is shown in Table 2, all the competences assessed in the rubric obtained significant better grades in P2 with respect to P1, as well as in the final grade of the Practicums, which was calculated through the arithmetic mean of all the competences in each Practicum. Table 3 presents the mean of each item and the comparisons between P1 and P2 (n = 61).

**Table 3:** Mean and standard deviation of each item of RECOPC-FIS II in P1 and P2. T values and significance level (p) for the comparison between P1 and P2 in each item.

Item	Practicum	Mean	SD	t	p
**C1**	P1	8.00	1.09	-3.778	.000
	P2	8.61	0.75		
**C2**	P1	8.21	0.73	-2.208	.031
	P2	8.48	0.76		
**C3**	P1	8.16	0.74	-2.974	.004
	P2	8.56	0.70		
**C4**	P1	8.14	0.84	-3.775	.000
	P2	8.65	0.72		
**C5**	P1	8.09	0.73	-5.160	.000
	P2	8.70	0.68		
**C6**	P1	8.06	0.76	-3.391	.001
	P2	8.48	0.68		
**C7**	P1	8.30	0.79	-3.105	.003
	P2	8.74	0.78		
**C8**	P1	8.29	0.85	-3.964	.000
	P2	8.77	0.68		
**C9**	P1	8.22	0.80	-2.904	.005
	P2	8.61	0.67		
**C10**	P1	8.65	0.93	-3.090	.003
	P2	9.01	0.72		
**C11**	P1	8.49	0.97	-3.669	.001
	P2	9.02	0.74		
**C12**	P1	8.48	0.89	-2.428	.018
	P2	8.85	0.82		
**C13**	P1	8.05	0.84	-4.269	.000
	P2	8.66	0.85		
**C14**	P1	8.15	0.85	-4.533	.000
	P2	8.74	0.70		
**Final Mean Grade**	P1	8.24	0.73	-4.232	.000
	P2	8.71	0.58		

The mean score for each item of the RECOPC-FIS II was between 7.97 for C1 (identification of the dysfunction or pathology) and 8.67 for C10 (interpersonal relationships) in P1; and 8.30 for C2 (physiotherapy diagnosis) and 8.93 for C10 (interpersonal relationships) in P2. The factor analysis showed the unidimensionality of RECOPC-FIS II (one single factor, explained 76.93% of the variance), as well as a high internal consistency (Cronbach’s α = 0.976). The RECOPC-FIS II tool was sensitive to detecting improvements in all competencies, comparing the scores of P1 with those of P2.

## Discussion

The obtained results demonstrate both the adequacy of the psychometric properties of the items and the reliability of RECOPC-FIS II. The different analyses conducted provide evidence of the validity of a 14-item structure and they show adequate statistical indices [39]. Similarly, a good index of internal consistency was obtained, resulting in a single scale that makes up the rubric [40].

Reliability and validity analyses that have been conducted during the design of the different clinical performance assessment tools in Physiotherapy included in the review by O’Connor et al. [38] showed poor results, which evidences the need for further research in this field. In the case of the RECOPC-FIS II, the values obtained in the analyses of the psychometric properties show the validity and reliability of the tool. In contrast, other instruments, such as the one proposed by Dogan et al. [36], only showed results from the analysis of inter-observer reliability. Regarding the number of participants with whom the analyses were conducted, in the study by Torres-Narváez [37], only 10 students and 3 clinical instructors in two care institutions were used, with ours being considerably larger in both the number of students and the number of clinical tutors. In contrast, one of the most complete analyses with the best results was published by Dalton et al. [12, 35], in which they showed internal consistency and validity values similar to ours.

Among the most important results of this study, this tool proved to be very sensitive to change, as can be observed from the analyses conducted with the 61 students from the 3^rd^ and 4^th^ year who used this rubric in two consecutive academic years. All the ratings of the 14 competences were higher in the P2 group with respect to P1. The changes in the health care provided in the different clinical settings through which our students pass in the different Practicums (P1 and P2) allow them to improve the competences acquired over time. These results could indicate that students are acquiring the necessary skills on a continuum, in such a way that, at the end of their studies, when they graduate, they achieve a consolidation of skills that qualifies them to enter real clinical practice, as suggested by Miller et al. in their study [41]. The competences outlined in the RECOPC-FIS II make this tool not only feasible and easy to use, but also have a significant educational impact, as they coincide with some of the proposals that appear in similar studies [38, 41, 42]. Thus, the challenge for educational institutions to assess clinical performance in the workplace determines a rarely studied feature of assessment tools [38]. The feasibility, pragmatism and lack of ambiguity provided by the use of our instrument will have a positive impact on both the rigorous assessment of students and the elimination of the subjective nature of observation-based assessment. Furthermore, the 14 evaluable criteria ensure an integral competence assessment for Health Sciences students in the real clinical context [12, 35, 37, 43–46] and particularly in what is considered in Royal Decree 592/2014 in the Degree of Physiotherapy in Spain [24, 45]. Moreover, they evaluate the most relevant transversal competences considered by other authors in the academic scope [17, 47]. Likewise, the results or our study are in line with those of Turpin et al. [48], which, although they are focused on Occupational Therapy, follow the competence evaluation line for Practicum students.

This rubric allows the student to recognize his/her advances, achievements and difficulties, analyze his/her evolution, and develop a critical and reflective attitude towards his/her educational process. Within each achievement level, the degree of task or behavior performance with respect to what must be evaluated in the student was clearly established, ensuring the maximum quality of care, evaluation objectivity and homogeneity among the different participants and care centers involved in the evaluation process. Incorporating these levels of achievement, which other tools have not contemplated [12, 35, 37], guarantees the optimum quality of the learning process and of the competence evaluation [49]. This process was explained to the students prior to beginning P1. Halfway through each rotation, the clinical tutor shows the student the items in which he/she must improve and how to do it. The constant feedback between the student and the clinical tutor allows both to stress on those aspects that require more attention and whose deficiencies would result in a lower grade and in a more deficient intervention for the patients. Verbal feedback can improve students’ learning and performance on clinical placements. Developing student feedback literacy may enhance feedback engagement and, therefore, learning outcomes [50]. This continuous and conscious learning could be a previous training for 4^th^-year students, which will allow them to further and better develop these skills, as is shown by the better grades obtained in P2 (although some of the competences may depend on the context and type of patient, as was previously mentioned), Enabling the acquirement of a skill assessment tool that is sensitive to change, which is a very important psychometric property. Further studies should be conducted in order to determine the degree of student satisfaction and usefulness of this assessment system.

In short, the present study emphasizes the importance of assessing transversal competences of the Physiotherapy Practicum using a valid and reliable tool such as the RECOPC-FIS II. Limitations or inconsistencies in the robustness of measurement tools may negatively affect clinical tutors’ decision making about the students they assess. Consequently, this could affect the practical preparation of graduates and even patient safety [51].

On the other hand, the acquisition of skills and qualities essential for the development of students’ professional life will be facilitated. It is of utmost importance for the acquisition of these skills and qualities to be extensible to all degrees in health sciences at the national and international level. Previous tools did not offer the possibility of being adapted to other degrees in health sciences, since they used competences specific to the profession [12, 35, 37]. Therefore, in line with the studies conducted by Bergsmann et al. [8], who proposed flexibility in assessment tools, the RECOP-FIS II would facilitate the adaptation of its items to the practicums of other degrees in health sciences. In addition, the completion of the assessment tool (online questionnaire) will ensure the quality of the assessment in clinical practices. It will allow determining, at all times, whether the development of transversal competences and their relationship with the contents of the different theoretical and practical courses correspond to the practicums of the degrees.

As a limitation of the present study, it is important to highlight the difference in the number of participants between the 3^rd^ and 4^th^ year students, since, during the first year of the study, it was only possible to collect data from students in P1, and data from students in the Practicum were only collected from 2 academic years.

In conclusion, RECOPC-FIS II proved to be a reliable and valid instrument for the evaluation of the transversal competences of the Practicum of Physiotherapy, providing useful information to both tutors and students. This tool would unify educational programs, promoting a shared and transparent common language when conducting assessments in health science courses. In line with other studies, this tool grants an essential role to the evaluation of the clinical practice in the training of physiotherapy students, being one of the few instruments published in Spanish [37].

## Supporting information

S1 Dataset Raw scores of all students. (XLSX)

S1 Appendix RECOPC-FIS II. (DOCX)
