# Supplementary material for: Competence assessment rubric in the Physiotherapy Practicum
Source: PLoS One. 2022 Feb 25;17(2):e0264120. doi: 10.1371/journal.pone.0264120 (PMC8880643; doi:10.1371/journal.pone.0264120)
Supplement: S1 Appendix — (DOCX) [file pone.0264120.s002.docx]

S1 Appendix. RECOPC-FIS II

| **Rubric for competence assessment in clinical physiotherapy practices**  (**RECOPC-FIS II)** | |
| --- | --- |
| **Initial information** | |
| E-mail |  |
| Rotation number | Check all that apply (1, 2, 3, 4) |
| Surname and name of student |  |
| Name of Clinical Practice Centre |  |
| Surname and name of clinical tutor |  |
| Scope of action | Mark one of these options:   - Traumatology and orthopaedics - Neurology - Paediatrics - Cardiovascular and respiratory - Geriatrics - Gynaecology and obstetrics |
| Interventions/actions carried out | Mark one of these options:   - Assessment and physical examination of the patient - Diagnosis in physiotherapy - Preparation of the clinical history - Physiotherapy treatment planning - Disease prevention and health promotion programmes - Evolution and/or discharge report |
| **Competence Assessment**  Next, we will ask them to rate from 1 to 10 the students’ performance in each of the transversal competences. To do this, take into account the description of each of the achievement levels. | |
| C1: Identification of the dysfunction or pathology | Information about achievement levels:   - From 1 to 4: Given the signs/symptoms presented, he/she is not able to identify the dysfunction or pathology. Does not reach basic conclusions. - From 5 to 6: The student identifies part of the signs/symptoms presented. He/she presents only one pathology and offers few hypotheses on the case. Basic, correct and consistent conclusions. - From 7 to 8: The student identifies most of the signs/symptoms presented. Generally, he/she offers a differential diagnosis by evaluating other similar pathologies. He/she justifies it superficially. Concrete and consistent conclusions. - From 9 to 10: He/she identifies almost all of the signs/symptoms presented. He/she almost always makes a differential diagnosis by evaluating other similar pathologies and justifies it in depth. Correct and consistent conclusions.   According to these levels of achievement, please indicate the final score (1–10). |
| C2: Physiotherapy diagnosis by assessing the functional status of the patient/user | Information about achievement levels:   - From 1 to 4: Unable to make a basic functional assessment of the patient with reliable procedures and instruments. - From 5 to 6: He/she is capable of making a basic functional assessment and uses some of the reliable procedures and instruments appropriately. Correct and consistent basic conclusions. - From 7 to 8: He/she is capable of making a complete functional assessment of the patient and uses reliable procedures and instruments appropriately. Correct and consistent basic conclusions. - From 9 to 10: He/she is capable of making a complete functional and comprehensive assessment of the patient. He/she uses reliable procedures and instruments in a very precise way, contrasting the data. Correct and consistent conclusions.   According to these levels of achievement, please indicate the final score (1–10). |
| C3: Therapeutic goals | Information about achievement levels:   - From 1 to 4: He/she does not have clear, coherent and feasible objectives. He/she does not take into account the patient’s own situation and expectations. - From 5 to 6: He/she establishes the objectives consistently but sometimes lacks evidence in the approaches. He/she does not always take into account the objectives set by the patient, the patient’s situation or stage of pathology. - From 7 to 8: He/she establishes the objectives in a coherent and evident way, prioritizing the needs. He/she considers the objectives set by the patient, the patient’s situation and the stage of the pathology. - From 9 to 10: He/she stands out in carrying out a comprehensive and hierarchical approach to the biopsychosocial needs of the patient, always taking into account the patient’s goals and expectations.   According to these levels of achievement, please indicate the final score (1–10). |
| C4: Integral treatment | Information about achievement levels:   - From 1 to 4: He/she does not contemplate the risks and contraindications. He/she is unable to come up with a basic and effective treatment plan. - From 5 to 6: He/she considers the risks and contraindications and raises and carries out a basic treatment plan, although incomplete. He/she is involved in the treatment of the patient and the patient’s relatives. - From 7 to 8: He/she considers the risks and contraindications and plans and carries out a complete treatment plan. He/she is involved in the treatment of the patient and the patient’s relatives. - From 9 to 10: He/she raises and carries out a comprehensive and individualized treatment plan according to the criteria of adequacy, validity and evidence. He/she is involved in the treatment of the patient and the patient’s relatives.   According to these levels of achievement, please indicate the final score (1–10). |
| C5: Treatment planning | Information about achievement levels:   - From 1 to 4: He/she does not specify therapies to achieve the therapeutic objectives set. He/she does not establish deadlines for achieving the objectives. - From 5 to 6: He/she specifies the therapies according to the symptoms presented but does not rank the order of application of daily treatments. Establishes short-term and sometimes medium- and long-term treatment. - From 7 to 8: He/she specifies and hierarchizes the application of part of the therapies according to the symptoms presented. Establishes short- and medium- and sometimes long-term treatment. - From 9 to 10: He/she specifies and hierarchizes the application of all the therapies according to the symptoms presented. He/she establishes short-, medium- and long-term treatment precisely.   According to these levels of achievement, please indicate the final score (1–10). |
| C6: Evaluation of the results | Information about achievement levels:   - From 1 to 4: He/she makes an insufficient evaluation of the results. He/she does not adapt the treatment plan based on the results obtained. - From 5 to 6: He/she makes an adequate assessment of the results obtained. Generally the student adapts the intervention plan to the new therapeutic objectives. He/she provides few alternatives or advantages and disadvantages. - From 7 to 8: He/she makes an adequate assessment of the results obtained. In most cases, the student adapts the therapeutic intervention plan, providing different alternatives and advantages and disadvantages. - From 9 to 10: He/she always adapts the intervention plan and evaluates the results obtained. He/she provides numerous alternatives, evaluating the advantages and disadvantages. The student provides arguments based on scientific evidence.   According to these levels of achievement, please indicate the final score (1–10). |
| C7: Technical and manual skills in the application of therapies (interventions) | Information about achievement levels:   - From 1 to 4: He/she is not capable of carrying out a large part of the techniques indicated in the process due to a lack of knowledge, autonomy and/or skill. The student does not show interest in those he/she does not know. - From 5 to 6: He/she does not know how to carry out one or several of the techniques proposed. The student has autonomy in the execution of most therapies, without any lack of skill, and considers the ergonomics. The student shows interest in those he/she does not know. - From 7 to 8: He/she performs most of the techniques proposed with skill, ergonomics and almost total autonomy. He/she adapts to the particularities of the patient. The student shows interest in those he/she does not know. - From 9 to 10: He/she carries out almost all of the techniques proposed with great skill, autonomy and ergonomics. He/she adapts to the particularities of the patient. The student shows interest in those he/she does not know.   According to these levels of achievement, please indicate the final score (1–10). |
| C8: Responsibility, patient care and suitability of clinical interventions | Information about achievement levels:   - From 1 to 4: Before acting, he/she barely reflects on the risks and consequences. His/her interventions are not always adequate when he/she poses a significant risk to the patient. The student does not detect or report critical incidents. - From 5 to 6: Before acting, he/she reflects on the risks and consequences. His/her interventions are always adequate when he/she poses a risk to the patient. In other types of interventions, the student is aware of his/her limitations and strives to improve. He/she detects and communicates critical incidents. - From 7 to 8: Before acting, he/she reflects deeply on the risks and consequences. His/her interventions are always appropriate when he/she poses a risk to the patient. The rest of the interventions are generally adequate and he/she strives to improve. He/she detects and communicates critical incidents. - From 9 to 10: Before acting, he/she reflects deeply on the risks and consequences. His/her interventions are always appropriate when he/she poses a risk to the patient. The other interventions are adequate and he/she argues them in a complete and coherent way. He/she detects and reports critical incidents.   According to these levels of achievement, please indicate the final score (1–10). |
| C9: Clinical reasoning | Information about achievement levels:   - From 1 to 4: He/she is not capable of identifying or evaluating the fundamental information. He/she does not reason logically or orderly in arguments. The student is not aware of his/her limitations and does not strive to improve. - From 5 to 6: He/she usually identifies and values the available information and reasons in a logical and orderly manner in arguments. He/she generally draws consistent conclusions (diagnosis, treatment and prognosis). The student is aware of his/her limitations and strives to improve. - From 7 to 8: Most of the time, he/she identifies and values all the fundamental information, reasoning logically and orderly in arguments to deduce coherent conclusions (diagnosis, treatment and prognosis). The student is aware of his/her limitations and strives to improve. - From 9 to 10: He/she always identifies and values all the fundamental information, always reasoning logically and orderly in arguments to deduce coherent conclusions (diagnosis, treatment and prognosis). The student is aware of his/her limitations and strives to improve.   According to these levels of achievement, please indicate the final score (1–10). |
| C10: Relationships | Information about achievement levels:   - From 1 to 4: He/she does not show verbal and non-verbal language skills or active listening attitudes. The student shows little sensitivity to the physical and emotional needs of the patient. He/she does not use dialogue to face conflict situations and his attitude is not very constructive. He/she is not aware of his emotionality or that of others and does not strive to improve. - From 5 to 6: He/she usually displays verbal and non-verbal language skills and active listening attitudes. In general, the student shows sensitivity towards the empathic patient’s physical and emotional needs. He/she generally confronts conflict situations with dialogue and positive and constructive attitudes. He/she is aware of his/her emotionality and that of others and strives for improvement. - From 7 to 8: He/she almost always shows verbal and non-verbal language skills and active listening attitudes. He/she almost always shows sensitivity towards the patient’s physical and emotional needs, with empathy. The student almost always faces conflict situations with dialogue and positive and constructive attitudes. He/she is aware of his emotionality and that of others and strives to improve. - From 9 to 10: He/she always displays verbal and non-verbal language skills and active listening attitudes. He/she always shows sensitivity towards the patient’s physical and emotional needs, with great empathy. The student always faces conflict situations with dialogue and positive and constructive attitudes. He/she is aware of his/her and others’ emotionality and strives to improve.   According to these levels of achievement, please indicate the final score (1–10). |
| C11: Oral communication with the patient, family and interdisciplinary team | Information about achievement levels:   - From 1 to 4: The student uses colloquial or inappropriate vocabulary. He/she provides confusing information and commands. He/she has difficulty relating effectively to the patient, family and team. He/she does not care about improvement. - From 5 to 6: The student normally uses the appropriate vocabulary for the cognitive state and cultural level of the patient, family and interdisciplinary team. Generally, he/she interacts effectively with the patient, family and team. He/she cares about continuous improvement. - From 7 to 8: The student almost always uses the appropriate vocabulary for the cognitive state and cultural level of the patient, family and interdisciplinary team. Generally, he/she is related in an efficient way with the patient, family and team. He/she cares about continuous improvement. - From 9 to 10: The student always uses the appropriate vocabulary for all contexts. He/she relates to the patient, family and team in a very effective way. He/she cares about continuous improvement.   According to these levels of achievement, please indicate the final score (1–10). |
| C12: Teamwork | Information about achievement levels:   - From 1 to 4: His/her presence and contributions to the team are irrelevant. The student intervenes only at the request of others. He/she does not respect the professional roles of team members. The student generates conflict in his/her interventions. - From 5 to 6: He/she is active and participates in group meetings. Sometimes the student makes fundamental contributions. Generally, he/she relates adequately with the rest of the members. He/she is aware of his/her limitations and strives to improve. - From 7 to 8: He/she is active and participative in group meetings. In general, the student makes fundamental contributions. He/she always relates appropriately with the rest of the members. He/she is aware of his/her limitations and strives to improve. - From 9 to 10: He/she is always very active and participative in group meetings. His/her contributions are always fundamental and very valuable and thus the quality of the team’s results improves. He/she always relates appropriately with the rest of the members.   According to these levels of achievement, please indicate the final score (1–10). |
| C13: Disease prevention and health promotion | Information about achievement levels:   - From 1 to 4: He/she is not capable of guiding the patient to adopt preventive and health promotion measures. - From 5 to 6: He/she guides the patient to adopt preventive and health promotion measures. - From 7 to 8: He/she generally guides the patient and family members to adopt preventive and health promotion measures. - From 9 to 10: He/she always guides the patient and his/her relatives to adopt preventive and health promotion measures.   According to these levels of achievement, please indicate the final score (1–10). |
| C14: Autonomous learning | Information about achievement levels:   - From 1 to 4: He/she does not have the minimum theoretical/practical knowledge that allows him/her to offer adequate quality of care. He/she is not aware of his/her limitations and does not strive to improve. The student barely goes deeper in the search for information that completes his/her theoretical/practical knowledge. - From 5 to 6: He/she shows a minimum level of theoretical/practical knowledge that allows him/her to offer adequate quality of care. - From 7 to 8: He/she shows an adequate level of theoretical/practical knowledge that allows him/her to offer adequate quality of care. The student provides basic and comprehensive information through various channels, incorporating new and useful knowledge. He/she is aware of his/her limitations and strives to improve. - From 9 to 10: He/she shows a high level of theoretical/practical knowledge that allows him/her to offer adequate quality of care. He/she excels at gaining insightful insights and building on the latest scientific evidence updates. The student is aware of his/her limitations and strives to improve.   According to these levels of achievement, please indicate the final score (1–10). |
| Other remarks: |  |
